# Peak oxygen uptake in relation to total heart volume discriminates heart failure patients from healthy volunteers and athletes

**DOI:** 10.1186/1532-429X-12-74

**Published:** 2010-12-16

**Authors:** Henrik Engblom, Katarina Steding, Marcus Carlsson, Henrik Mosén, Bo Hedén, Torsten Buhre, Björn Ekmehag, Håkan Arheden

**Affiliations:** 1 Dept of Clinical Physiology, Lund University and Skåne University Hospital, Lund, Sweden; 2 Dept of Sport Sciences, Malmö University, Malmö, Sweden; 3 Dept of Cardiology, Lund University and Skåne University Hospital, Lund, Sweden

## Abstract

**Background:**

An early sign of heart failure (HF) is a decreased cardiac reserve or inability to adequately increase cardiac output during exercise. Under normal circumstances maximal cardiac output is closely related to peak oxygen uptake (VO_2_peak) which has previously been shown to be closely related to total heart volume (THV). Thus, the aim of this study was to derive a VO_2_peak/THV ratio and to test the hypothesis that this ratio can be used to distinguish patients with HF from healthy volunteers and endurance athletes. Thirty-one patients with HF of different etiologies were retrospectively included and 131 control subjects (60 healthy volunteers and 71 athletes) were prospectively enrolled. Peak oxygen uptake was determined by maximal exercise test and THV was determined by cardiovascular magnetic resonance. The VO_2_peak/THV ratio was then derived and tested.

**Results:**

Peak oxygen uptake was strongly correlated to THV (r^2 ^= 0.74, p < 0.001) in the control subjects, but not for the patients (r^2 ^= 0.0002, p = 0.95). The VO_2_peak/THV ratio differed significantly between control subjects and patients, even in patients with normal ejection fraction and after normalizing for hemoglobin levels (p < 0.001). In a multivariate analysis the VO_2_peak/THV ratio was the only independent predictor of presence of HF (p < 0.001).

**Conclusions:**

The VO_2_peak/THV ratio can be used to distinguish patients with clinically diagnosed HF from healthy volunteers and athletes, even in patients with preserved systolic left ventricular function and after normalizing for hemoglobin levels.

## Introduction

Heart failure (HF) is a complex syndrome associated with a variety of etiologies and clinical presentations [1] which implies a major diagnostic challenge. The accuracy of diagnosis by clinical means alone is often inadequate [2,3]. According to the definition of HF, patients should exhibit typical symptoms or signs such as breathlessness or fatigue at rest or during exercise, pulmonary congestion or ankle swelling, and objective evidence of cardiac dysfunction at rest [1]. However, ACC/AHA have identified 4 stages (A-D) with emphasis on the evolution and progression of HF, where stage A defines patients who are at high risk for developing HF but has no structural disorder of the heart and no signs or symptoms of HF [4]. Thus, there is a need for diagnostic methods that can objectify early signs of decreased cardiac performance in order to optimize management and treatment to prevent or delay progression of the disease and consequently improve patient prognosis.

Cardiac performance can be assessed by maximal exercise testing with measurements of respiratory gas exchange to determine peak oxygen uptake (VO_2_peak), shown to be closely correlated to maximal cardiac output [5,6]. This method is predominantly used in evaluation of patients with established HF under consideration for heart transplantation [7,8]. Experimental animal studies [9,10] and a recent study in humans [11] have shown that VO_2_peak is closely correlated to the total heart volume (THV). Hence, a physiologically enlarged heart has a proportionally increased VO_2_peak. A decreased cardiac reserve and inability to increase cardiac output to meet the metabolic requirements during exercise might be the first sign of HF [12].

Cardiovascular magnetic resonance (CMR) is well suited for determination of the THV, given the completely three dimensional nature of the technique. CMR is also considered the gold standard for determination of cardiac function and dimensions [13].

Therefore, the aim of this study was to use maximal exercise testing with measurements of respiratory gas exchange and CMR to derive a VO_2_peak/THV-index as a novel measure of the cardiac reserve and to test if this can be used to distinguish patients with HF from healthy volunteers and endurance athletes with physiologically enlarged hearts.

## Methods

### Study population and design

This study follows the Declaration of Helsinki and was approved by the regional ethics committee. All study subjects gave their informed consent to participate in the study.

Thirty-one patients (5 females) who had been clinically diagnosed with HF with varying etiology were retrospectively enrolled and compared with 60 healthy volunteers (20 females) from the local community and 71 elite athletes (30 soccer players [12 females], 23 European handball players [12 females] and 18 triathletes [6 females]) prospectively enrolled in the study. All underwent both CMR and maximal incremental exercise testing on ergometer cycle, with measurement of respiratory gas exchange, within a month for the patients and within a week for the healthy volunteers and athletes. Hemoglobin level was assessed within 2 weeks of the exercise test in patients and on the day of the exercise test in healthy volunteers and athletes who gave their consent for blood sampling.

### CMR and analysis

CMR was performed on a 1.5 T system (Philips Intera CV; Philips, Best, The Netherlands) with a cardiac synergy coil. All subjects were placed in supine position. Cine short-axis gradient-recalled echo images covering the entire heart from base of the atria to the ventricular apex were acquired using a steady state free precession sequence - slice thickness = 8 mm, field-of-view = 340 mm, repetition time = 3.14 ms, echo time = 1.58 ms.

For viability assessment in the patients, late gadolinium enhancement (LGE) images were acquired 20--30 minutes after intravenous injection of an extracellular gadolinium-based contrast agent (gadoteric acid, Gd-DOTA, 0.2 mmol/kg, Guerbet, Gothia Medical AB, Billdal, Sweden). An inversion-recovery (IR) sequence was applied in the corresponding short-axis planes as for the cine images. Typical IR sequence parameters were: slice thickness = 8 mm, repetition time = 3.9 ms, echo time = 1.2 ms, in-plane resolution = 1.5 × 1.5 mm and flip angle = 15° with acquisition every heartbeat. The inversion time, typically 200--300 ms, was manually adjusted to null the signal from remote myocardium [14].

#### Volumetric measurements

**Figure 1:**
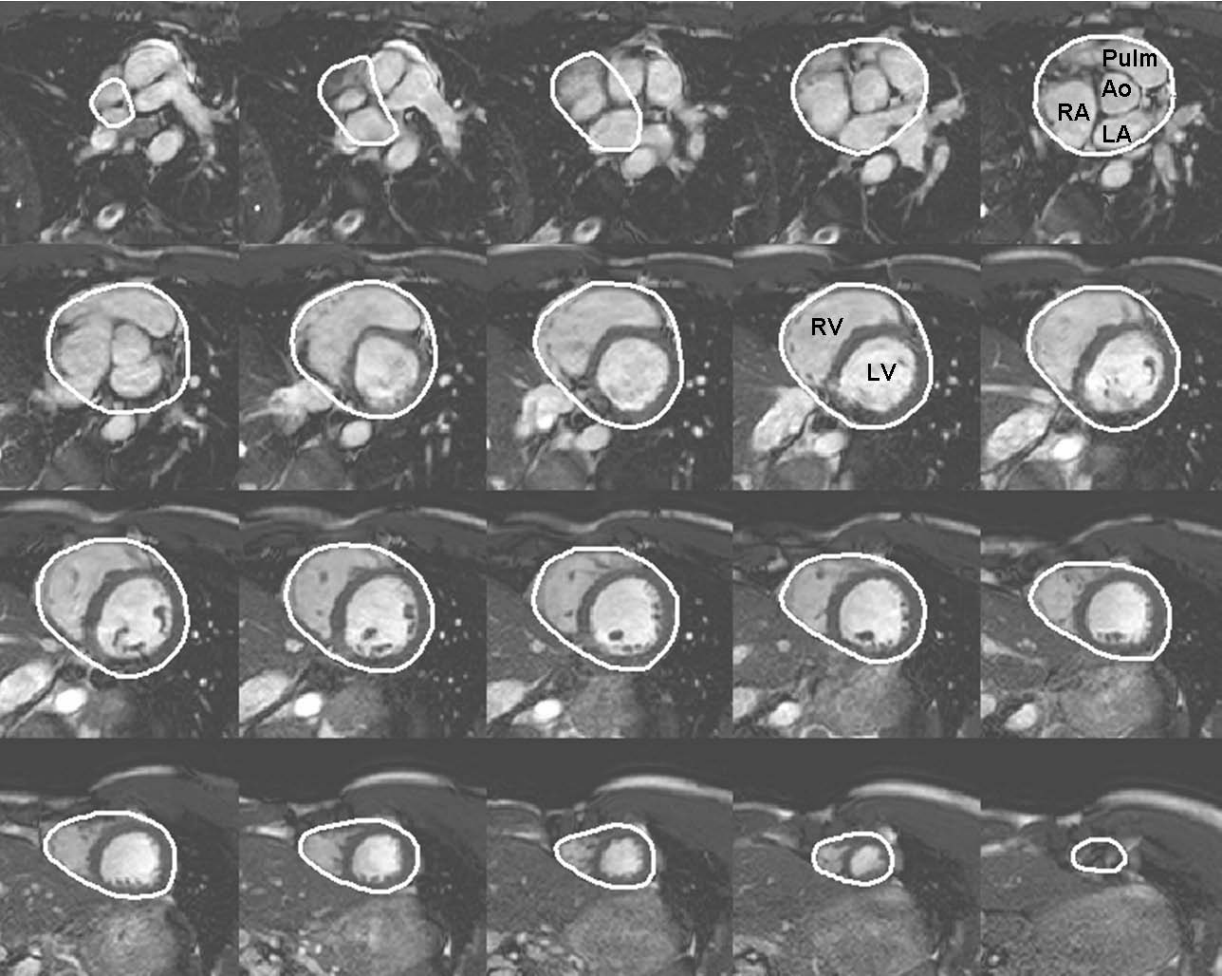
**Delineation of total heart volume**. Example of delineation of the pericardium from short-axis cardiac magnetic resonance images enabling determination of the total heart volume. The base is seen in the upper left corner and apex in the lower right corner. Ao = ascending aorta, LA = left atrium, LV = left ventricle, Pulm = pulmonary artery, RA = right atrium, RV = right ventricle.

Total heart volume was measured in the cine short-axis images by planimetry as previously described [15]. In short, THV was defined as the volume of all structures within the pericardium, including myocardium, blood pool, atria and pericardial fluid. This also includes the proximal parts of the great vessels covered by the pericardium. A region of interest was manually drawn around the pericardial border in all the short-axis images of the heart in end-diastole (Figure 1) using a freely available software (Segment 1.697; http://segment.heiberg.se) [16]. The summed area was then multiplied with slice thickness to obtain THV. The volumetric method used to determine THV has previously been applied in studies of cardiac pumping mechanics [15,17-21].

Furthermore, endocardial borders of the left ventricular (LV) myocardium were manually delineated in end-diastolic and end-systolic short-axis images, enabling calculation of LV end-diastolic and end-systolic volumes (LVEDV and LVESV) as well as LV ejection fraction (LVEF). In addition, the epicardial borders of the left ventricle were delineated for determination of LV mass (LVM).

#### Viability assessment

Myocardial scar/fibrosis was assessed by manual delineation of the hyperenhanced myocardium in the LGE short-axis images.

### Exercise test with respiratory gas analysis

A maximal incremental exercise test was performed using an electronically braked cycle ergometer (Siemens Ergomed 940, Upplands Väsby, Sweden) with respiratory gas analysis equipment (Oxycon Champion, Jaeger, Hochberg, Germany). The test protocols were chosen to yield exercise duration of approximately 8-12 minutes, yielding starting stress between 10-150 W with 10-30 W increment per minute. Peak oxygen uptake was defined as the highest value reached at the end of exercise and expressed both as ml min^-1 ^(VO_2_peak) and ml min^-1 ^kg^-1 ^(VO_2_peak_kg_).

A 12-lead ECG was acquired throughout the test. Blood pressure was measured by sphygmanometer in the supine position before exercise, every two minutes during exercise on the ergometer cycle and in the supine position at rest after exercise.

### VO_2_peak/THV ratio

A ratio between VO_2_peak and THV was derived. Furthermore, the ratio was also normalized to hemoglobin levels to partly compensate for differences in oxygen delivery capacity in peripheral tissue in the presence of anemia. The VO_2_peak/THV ratio was also compared to levels of plasma amino terminal pro-brain natriuretic peptide (pro-BNP) for the patients who had a pro-BNP taken within 4 months of the exercise test.

### Statistical analysis

Values are shown as mean ± standard deviation. Univariate linear regression analysis was used to assess the correlation between VO_2_peak and THV as well as body weight. Unpaired T-test was used to assess differences between variables where normal distribution was assumed. The Mann-Whitney test was used to assess differences where normal distribution could not be assumed. Forward stepwise multivariate regression analysis was performed to assess the independent predictive values of THV, LVEF, LVEDV, VO_2_peak, VO_2_peak_kg _and the VO_2_peak/THV ratio for presence of HF. Receiver operating characteristic (ROC) analysis was performed to test the ability of the VO_2_peak/THV ratio to distinguish between HF patients and controls. All statistical analysis was performed using SPSS 16.0 (Chicago, IL, USA). A p-value of <0.05 was considered statistically significant.

## Results

Subject characteristics are seen in Table 1. Twelve patients had been diagnosed with dilated cardiomyopathy, five patients with ischemic cardiomyopathy, three patients with suspected myocardial hemocromatosis, two patients with hypertrophic cardiomyopathy, two patients with congenital heart disease, two patients with constrictive cardiomyopathy, one patient with post-partum cardiomyopathy, one patient with drug-induced cardiomyopathy, and three patients with HF of unknown cause. In 16% (5/31) of the patients there were signs of scarred/fibrous myocardium on the LGE images. Hemoglobin levels were assessed the same day as the exercise test in 65% (39/60) of the healthy volunteers and in 73% (52/71) of 71 athletes (hemoglobin levels were not assessed in the triathletes) and within 2 weeks of the exercise test in 97% (30/31) of the patients.

**Table 1:** Patient characteristics

	**Healthy volunteers**	**Athletes**	**Patients**
	
**Number**	**n = 60**	**n = 71**	**n = 31**
Gender (M/F)	40/20	41/30	26/5
Age (years)	35 ± 11	26 ± 7***	54 ± 12***
Weight (kg)	75 ± 11	75 ± 11	80 ± 15
Height (m)	1.77 ± 0.08	1.78 ± 0.08	1.74 ± 0.08
SBP (mmHg)	127 ± 9	126 ± 9	107 ± 12***
DBP (mmHg)	74 ± 8	72 ± 8	69 ± 9**
VO_2_peak (ml min^-1^)	3102 ± 696	3866 ± 747***	1309 ± 430***
VO_2_peak_kg _(ml min^-1 ^kg^-1^)	41 ± 7	52 ± 7***	16 ± 5***
LVEDV (ml)	183 ± 31	223 ± 39***	280 ± 132***
LVM (g)	105 ± 25	130 ± 34***	177 ± 87***
THV (ml)	793 ± 128	947 ± 169***	1201 ± 460***
LVEF (%)	62 ± 6	58 ± 6**	37 ± 22***

DBP = diastolic blood pressure, LVEDV = left ventricular end-diastolic volume, LVEF = left ventricular ejection fraction, LVM = left ventricular mass, SBP = systolic blood pressure, THV = total heart volume, VO_2_peak = peak oxygen uptake, **p < 0.01, ***p < 0.001 compared to the healthy volunteers

The inter-observer variabililty for measuring THV was -5±37 ml (r^2 ^= 0.96, p < 0.001).

### Peak oxygen uptake vs cardiac characteristics and body weight

**Figure 2:**
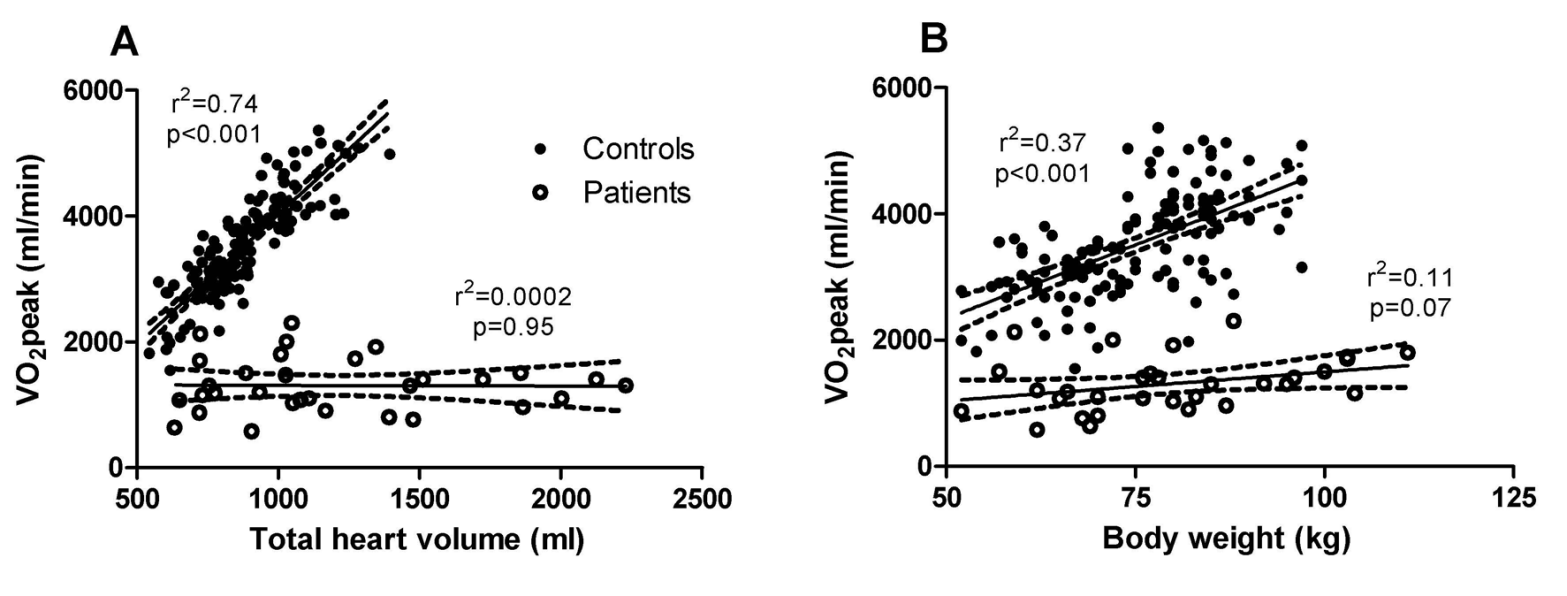
**Peak oxygen uptake in relation to total heart volume and body weight**. Peak oxygen uptake (VO_2_peak) in comparison to **A) **total heart volume (THV) and **B) **body weight. Filled circles denote control subjects, including healthy volunteers and athletes and open circles denote heart failure patients. Solid lines represent the regression line and dashed lines the 95% confidence interval. In the control group there was a strong correlation between VO_2_peak and THV. Even though there was also a significant correlation between VO_2_peak and body weight, there was a considerable variation of VO_2_peak for a given body weight.

There was a strong correlation (r^2 ^= 0.74, p < 0.001) between VO_2_peak and THV for the control subjects (healthy volunteers and athletes) but no correlation (r^2 ^= 0.0002, p = 0.95) for HF patients (Figure 2A). Figure 2B shows only a moderate correlation (r^2 ^= 0.37, p < 0.001) between VO_2_peak and body weight in the control subjects with a considerable variation in VO_2_peak for a given body weight.

**Figure 3:**
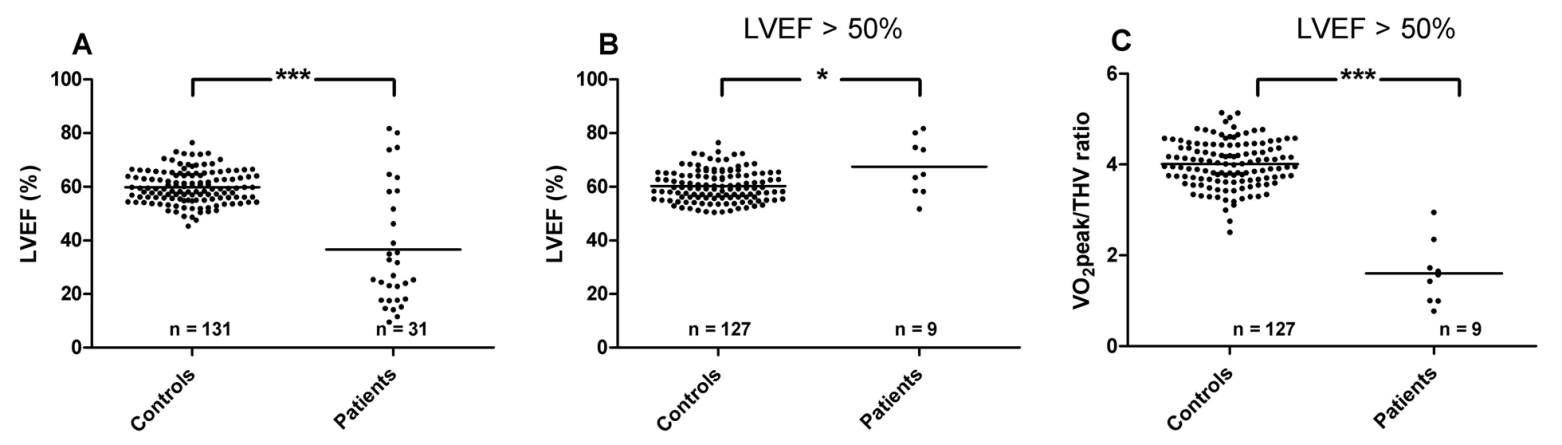
**Left ventricular ejection fraction in relation to VO_2_peak/THV ratio**. Left ventricular ejection fraction (LVEF) for **A) **all subjects and **B) **control subjects and heart failure patients with LVEF > 50% and **C) **VO_2_peak/THV ratio for the control subjects and heart failure patients with LVEF > 50%. In the heart failure patients with LVEF > 50% there was a significantly lower VO_2_peak/THV ratio despite a slightly higher LVEF compared to the control subjects. The solid line represents the mean value. * p < 0.05 and *** p < 0.001

Even though a significant difference in LVEF was found between the control subjects and the HF patients (p < 0.001; Figure 3A), there was a significant overlap with 29% (9/31) of the HF patient examinations showing LVEF > 50%. Figure 3B shows a comparison between control subjects and HF patients with LVEF > 50%, where the HF patients had a slightly higher LVEF (p = 0.04). Despite the higher LVEF in these patients, the VO_2_peak/THV ratio was significantly lower compared to the control subjects (p < 0.001; Figure 3C).

### VO_2_peak/THV ratio

**Figure 4:**
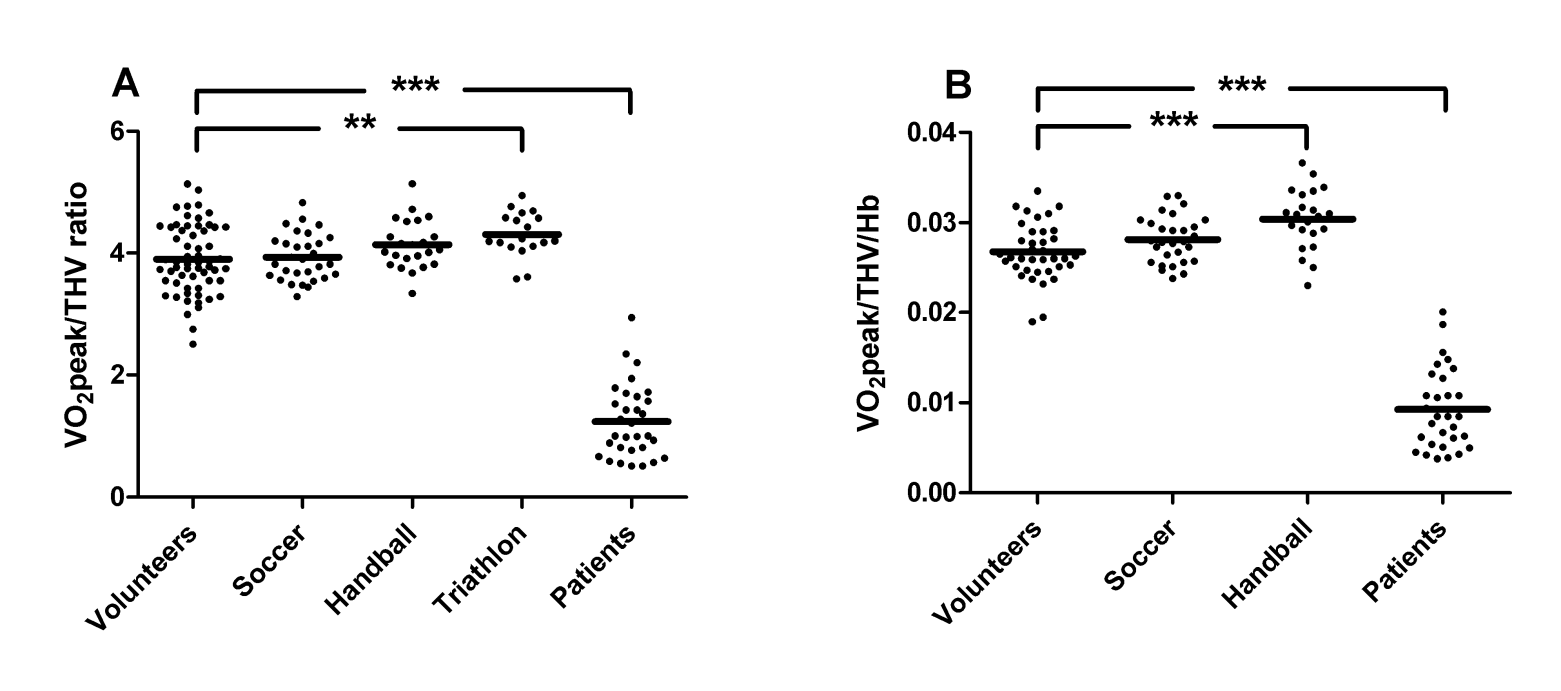
**VO_2_peak/THV ratio in heart failure patients and control subjects**. VO_2_peak/THV ratio in heart failure patients compared to healthy volunteers and different groups of athletes. The VO_2_peak/THV ratio was significantly lower in the heart failure patients compared to the control groups (**A**). This relationship remained unchanged after normalizing the results to hemoglobin levels (**B**). The solid line represents the mean value. ** p < 0.01 and *** p < 0.001 compared to healthy volunteers.

Figure 4A shows the VO_2_peak/THV ratio for each subject group. The HF patients showed a significantly lower VO_2_peak/THV ratio (p < 0.001) than the other subject groups, even after normalizing for hemoglobin levels (p < 0.001, Figure 4B).

**Figure 5:**
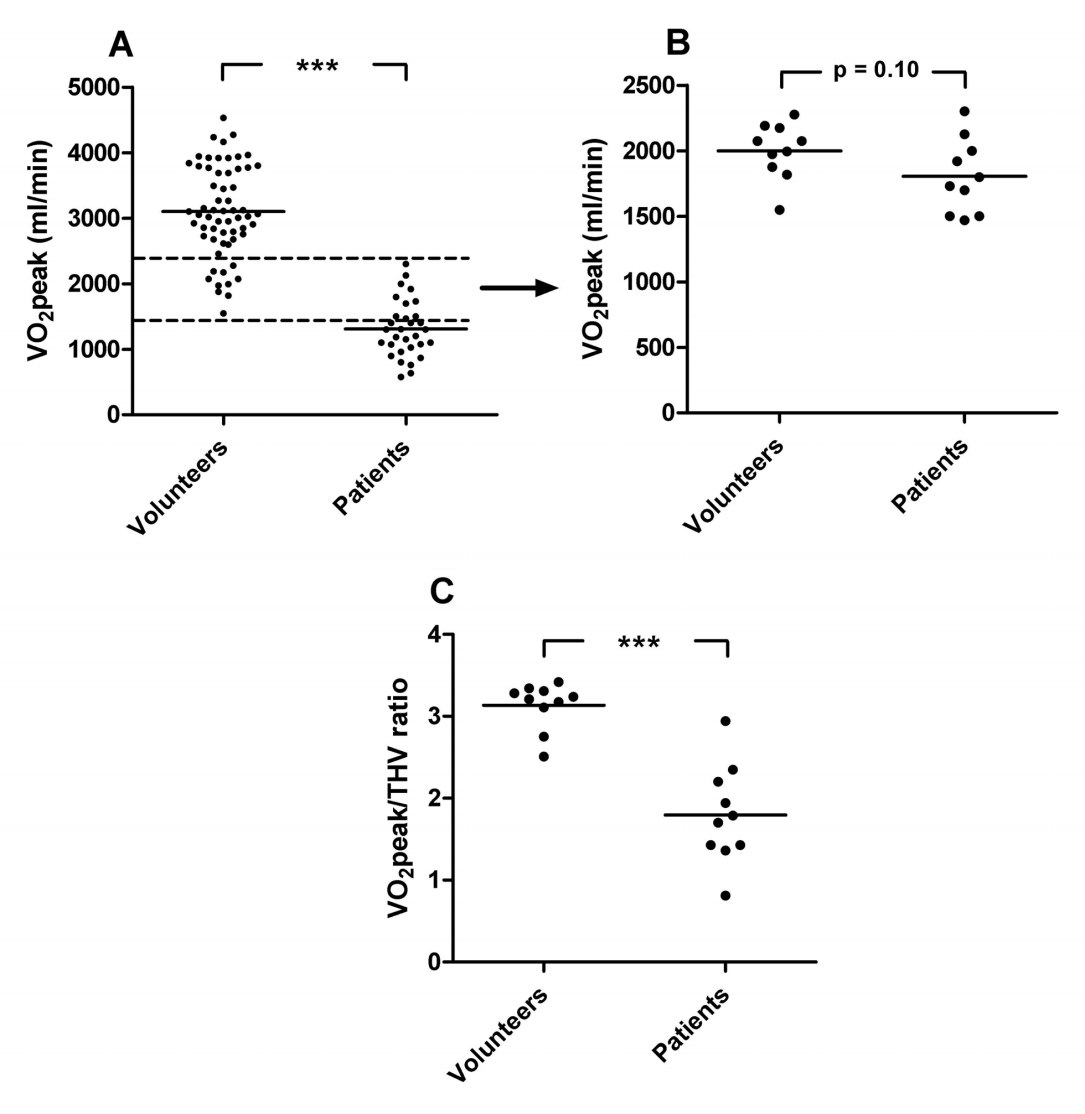
**Peak oxygen uptake in comparison to VO_2_peak/THV ratio**. Peak oxygen uptake (VO_2_peak) for the healthy volunteers and the HF patients (**A**) which showed a considerable overlap (**B**). The VO_2_peak/THV ratio was, however, significantly lower in the 10 heart failure patients compared to the 10 healthy volunteers with overlapping VO_2_peak (**C**). Dashed lines indicate the overlap between the two groups and the solid lines represents mean values. *** p < 0.001

When dividing the different subgroups with regard to VO_2_peak alone, there was still a significant difference between healthy volunteers and HF patients (3102 ± 696 vs 1309 ± 430 ml/min; p < 0.001). However, there was a considerable overlap between the two groups (Figure 5A). Ten control subjects and 10 HF patients had overlapping VO_2_peak (Figure 5B). The VO_2_peak/THV ratio, however, differed significantly between these two groups (p < 0.001) (Figure 5C). The LVEF for the overlapping healthy volunteers did not differ significantly from the overlapping HF patients (42 ± 25% vs 61 ± 5%; p = 0.11), whereas THV was significantly larger for the latter (640 ± 66 vs 1092 ± 336 ml/min; p < 0.001).

In 68% (21/31) of the HF patients a pro-BNP was taken 11 ± 25 days from the exercise test. There was a significant correlation between decreasing VO_2_peak/THV ratio and increasing pro-BNP (r^2 ^= 0.42, p = 0.002).

**Figure 6:**
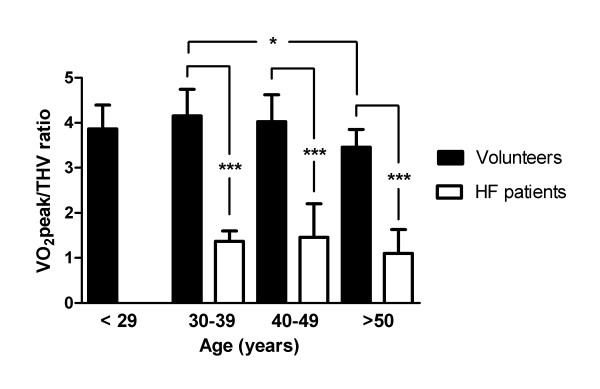
**VO_2_peak/THV ratio in different age groups**. VO_2_peak/THV ratio in healthy volunteers and in heart failure patients in different age groups. Even though there was a slight decrease in VO_2_peak/THV ratio with increasing age in the healthy volunteers, VO_2_peak/THV ratio remained significantly higher in the healthy volunteers compared to the heart failure patients. * p < 0.05 and *** p < 0.001

Furthermore, the VO_2_peak/THV ratio was the only independent predictor of HF (p < 0.001; Table 2). The area under the curve (AUC) for the VO_2_peak/THV ratio to diagnose HF was 1.0. With a cut-off value of 3.0, the VO_2_peak/THV ratio had a sensitivity of 100% and a specificity of 97%. Even though there was a slight decrease in VO_2_peak/THV ratio with increasing age, there was a significant difference between patients and healthy volunteers for all age groups (Figure 6).

**Table 2:** Forward stepwise multivariate regression analysis for prediction of heart failure

	Beta-value (r-value)	p-value
**VO_2_peak/THV ratio**	**-0.90**	**< 0.0001**
**Excluded variables**	**Partial correlation**	

THV	0.03	0.74
LVEDV	0.05	0.53
LVEF	-0.10	0.23
VO_2_peak (ml min^-1^)	0.03	0.74
VO_2_peak_kg _(ml min^-1 ^kg^-1^)	-0.14	0.09

LVEDV = left ventricular end-diastolic volume, LVEF = left ventricular ejection fraction, THV = total heart volume, VO_2_peak = peak oxygen uptake

## Discussion

This study shows that a VO_2_peak/THV ratio can be used to distinguish patients with HF from healthy volunteers and athletes, even in patients with preserved systolic LV function and after normalizing for hemoglobin levels.

### Clinical use of the proposed VO_2_peak/THV ratio

It is expected that the VO_2_peak/THV ratio will drop in the presence of HF, since cardiac output, and consequently VO_2_peak, is disproportional to the metabolic requirements of the peripheral tissue in the situation of a failing heart. However, when and in which stage of HF development the VO_2_peak/THV ratio decreases is not known. A decreased VO_2_peak/THV ratio could potentially be seen in patients with no symptoms at rest and in the absence of significant structural cardiac abnormalities.

Currently, maximal exercise test with analysis of respiratory gases and analysis of VO_2_peak is predominantly used in patients with terminal HF under consideration for heart transplantation [7]. The present study suggests that assessment of VO_2_peak in relation to THV for determination of a VO_2_peak/THV ratio can be of potential use even in non-terminal and early stage HF, maybe even before the patient exhibit symptoms, such as in stage A HF [4]. Another possible application of a VO_2_peak/THV ratio is in management of patients with a history of endurance training and a sudden change in exercise capacity. These patients might perform within normal limits during a maximal exercise test with analysis of respiratory gases when compared to age and gender matched controls. However, in relation to THV the working capacity might be decreased which would be detected by a low VO_2_peak/THV ratio as an indicator of early stage HF. This is supported by the findings that the healthy volunteers and the HF patients with overlapping VO_2_peak showed a significant difference in VO_2_peak/THV ratio with almost no overlap, indicating that the ratio has additional diagnostic value. Furthermore, LVEF was not statistically significant different between the two overlapping groups. Thus, VO_2_peak/THV provides diagnostic information beyond LVEF.

Even though there was a significant difference between control subjects and HF patients with regard to LVEF in the present study, almost 30% of the patients had normal LVEF. However, these patients had significantly lower VO_2_peak/THV ratio than the control subjects with almost no overlap. Thus, VO_2_peak/THV ratio may also be used as a marker of HF in patients with preserved systolic LV function.

Heart failure is often described based on clinical symptoms as being mild, moderate, or severe. Mild HF is used for patients with no significant physical limitations due to dyspnoea or fatigue and severe HF is used for patients who are markedly symptomatic with a need for frequent medical attention. Moderate HF is used for the remaining patients. For clinical management of HF patients and for randomized clinical trials, the New York Heart Association (NYHA) functional classification [22,23] is often employed. However, the accuracy of diagnosing HF by clinical means alone is often inadequate [2,3]. Hence, there is a need for objective measures of cardiac performance in order to determine the efficiency of different therapeutic strategies in randomized clinical trials.

The clinical use a VO_2_peak/THV ratio could potentially complement commonly used cardiac diagnostic biomarkers such as ECG and biochemical markers. A commonly used biochemical marker for severity of HF is pro-BNP, which has been shown to correlate with the severity of HF [24,25]. In the present study, the VO_2_peak/THV ratio was shown to correlate with pro-BNP, indicating that the ratio can potentially serve as a measure for severity of HF.

### Normalization of peak oxygen uptake to cardiac dimension rather than body weight

Lower limits for VO_2_peak are usually determined in relation to body weight and used to determine if a patient is suitable for heart transplantation or not [26,27]. However, the present study show a considerable variability in VO_2_peak for a given body weight (Figure 2B). Total heart volume, however, was shown to be closely correlated to VO_2_peak in healthy volunteers and athletes but not in HF patients. Thus, for the non-failing heart, the VO_2_peak/THV ratio is preserved in the presence of a physiologically enlarged heart, whereas for the failing heart the VO_2_peak/THV ratio is not preserved as THV increases due to pathological enlargement of the heart. This suggests that VO_2_peak should be normalized to THV and not to body weight. This is in line with a recent paper by Saltin et al [28] and experimental animal studies [9,10] showing that VO_2_peak is determined primarily by cardiac dimension and not peripheral factors such as the efficiency of oxygen uptake in peripheral tissue [29]. This is also supported by the present study showing that a VO_2_peak/THV ratio is significantly lower in HF compared to healthy volunteers and athletes even after normalizing for hemoglobin levels.

### Determination of total heart volume

In the present study THV was determined by CMR, which is considered the gold standard for assessing cardiac dimensions and function [13]. In situations when CMR is not available or is contraindicated, i.e. in patients with biventricular pacing, THV could potentially also be determined by low dose-cardiac CT, preferably gated. However, non-gated examinations would also be possible if CT thorax is performed for other reasons, since THV varies little during the cardiac cycle [15,17,18]. Three dimensional echocardiography might potentially also be used to determine THV, although this technique may suffer from incomplete coverage of the tissue within the pericardium. By using the THV, enlargement of all cardiac chambers including the atria is considered. Enlargement of the left atrium has been shown to provide prognostic value in HF patients with normal LVEF [30].

### Limitations

The results of the present study should be interpreted in the light of some limitations. The number of patients in the present study is limited and the patients were retrospectively included with clinically diagnosed HF and clinically determined etiology. Thus, no specific HF inclusion criteria based on biochemical markers, ECG or echocardiographic variables were applied. Furthermore, the patients had different etiologies and different stages of HF. However, this pilot study can be considered a proof of the concept study for determining a VO_2_peak/THV ratio as an objective measure of cardiac capacity, independent of HF etiology.

Due to symptoms during the exercise test, HF patients might not reach VO_2_peak representative of the individual's actual maximal oxygen uptake capacity. For this reason VO_2_peak is referred to as peak and not maximal oxygen uptake in the present study.

There was a significant difference in mean age between the patient population and the control groups. However, the slight decrease in VO_2_peak/THV ratio found with increased age in the healthy volunteers (21-65 years) is negligible compared to the decrease in VO_2_peak/THV ratio found in the HF patients.

Since VO_2_peak can not be obtained in HF patients with acute decompensation, the VO_2_peak/THV ratio can not be used in these patients. The VO_2_peak/THV ratio might, however, be of significant benefit when the HF diagnosis is uncertain, such as in the situation of stage A HF or if the progression of the disease is less clear.

Furthermore, the prognostic value of the VO_2_peak/THV ratio also remains to be determined in prospective follow-up studies of different patient groups with established HF or at risk for developing HF.

## Conclusions

The VO_2_peak/THV ratio can be used to distinguish patients with known HF from healthy volunteers and athletes, even in patients with preserved systolic LV function and after normalizing for hemoglobin levels.

## Competing interests

The authors declare that they have no competing interests.

## Authors' contributions

HE, KS, MC, and HA have made substantial contributions to conception and design of the study. HE, KS, MC, TB, BE, HM, and BH, were responsible for acquisition of data. HE, KS, MC, BE, and HA were responsible for analysis and interpretation of the data. All authors have been involved in drafting the manuscript and revising it critically for important intellectual content. Furthermore, all authors have given final approval of the current version to be published.
